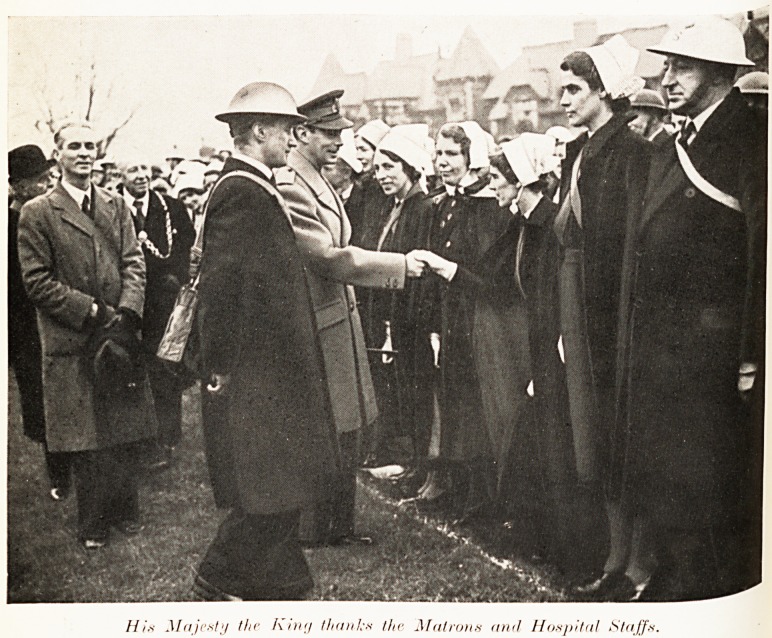# The Story of the Bristol Hospitals

**Published:** 1942

**Authors:** 


### THE STORY OF THE BRISTOL HOSPITALS.

---

THE following account of the Bristol Hospitals during the 1940–41 series of air raids, is reprinted from *Bristol Under Blitz* (Arrowsmith, 2s. 6d. net), by permission of the author, Alderman T. H. J. Underdown, and the Lord Mayor's War Services Council. Some account of the hospital experiences during blitzes would have appeared before in the *Journal* but for censorship restriction. Now that these have been relaxed for Alderman Underdown's book, we welcome the opportunity of giving the story in the words of the first official account of Bristol's Air Raids, and express our thanks to the Author, the War Services Council and the publishers.

Bristol is well served by an efficient Hospital system. The largest voluntary Hospitals are the Royal Infirmary, the General Hospital and the Children's Hospital, with the Cossham Memorial Hospital in the Kingswood area. The Southmead Hospital was established and is maintained by the Health Department of the City Corporation.* Winford Orthopædic Hospital is situated in Somerset, about five miles south of the City. Specialist treatment is given at the Homœopathic and Eye Hospitals. The Ham Green Hospital and Sanatorium, also in the County of Somerset, is used by the City Corporation for isolation cases. All these institutions rendered conspicuous service for those severely injured through enemy action.

Civilians treated in the Hospitals and listed as seriously injured included many men, women and children. To these were added men of the Services who met with serious injury whilst in discharge of their duties. A glimpse at a few incidents which occurred at these institutions gives some inkling of the stress and strain borne by the staffs during the period of the major raids upon this City.

The Bristol General Hospital suffered much damage, but not a single patient nor a member of the staff was seriously injured, which was a great consolation considering the damage sustained by the building. During the period of heavy attacks upon the City many high explosive bombs were dropped in close proximity to the Hospital, shattering windows and blowing the doors to pieces. On 2nd December

* EDITOR'S NOTE.—Southmead Hospital has actually a far longer history. In 1696 the Bristol Corporation of the Poor established St. Peter's Hospital in the old Mint. This hospital outgrew its premises and underwent several removals without any break in the continuity of its service. In 1915 the Guardians of the Poor had just completed the building of a new hospital at Southmead. This was occupied as a Military Hospital until 1919, when it was handed back to the Guardians and opened as a Poor Law hospital. Under the 1929 Act the hospital passed into the management of the Health Committee of the City Corporation. Thus Southmead Hospital and its forerunners cover an unbroken period of nearly 250 years. St. Peter's Hospital was the first post-reformation hospital founded in England.

*The Prime Minister, with Mr. Menzies and Mr. Winant, outside the University—Easter Saturday, 1941.*

*H.R.H. the late Duke of Kent, with the Regional Commissioner, General Sir Hugh Elles.*

*His Majesty the King thanks the Matrons and Hospital Staffs.*

many heavy calibre bombs fell within 50 yards of the buildings, blew in most of the windows and threw masses of mud and debris into the wards and rooms adjoining. Fortunately all the patients had previously been moved to the basement shelter before the electric current, water and gas supplies failed. The weather was bitterly cold at this time and the conditions prevailing inside the Hospital were dreadful, but the staff gallantly carried on and saw to the safe evacuation of the patients. Weeks elapsed before temporary repairs could be effected to allow the wards to be used again, but the casualty and out-patient services were not interrupted.

No sooner had the Hospital commenced to function again when, during an intensive raid in January, a serious fire occurred through quantities of incendiary bombs, 16 in one tray, falling on the roof. The staff and members of the Auxiliary Fire Service stationed at the Hospital were unable to deal with this before the night nurses' and the maids' quarters on the top floor were furiously blazing. The fire rapidly spread to the tower, the dome of which was destroyed, together with the contents of the night nurses' and the maids' rooms and three wards below. At the same time high explosive bombs were dropped close at hand and the blast from these caused still more damage. There was a good supply of water available, pumped from the dock by motor pumps, and it was solely due to this that the Hospital was saved from complete destruction.

The conditions prevailing in the Hospital during the fire and for some time afterwards were well-nigh beyond description. Water and filth cascaded down staircases, lift-shafts and air ducts and through ceilings. Water was everywhere.

The staff was wonderful, and stood bravely up to the difficulties and dangers. The Matron, Miss A. C. Robins, has since been awarded the O.B.E. for the noble example she set to her staff throughout. Naturally it took some time to recover from this onslaught in the damp and sour atmosphere which prevailed, but all stuck to it in spite of the absence of electricity or gas for days.

An underground operating theatre unit was provided later, where the surgeons worked in comparative safety, with wards adjoining for air-raid casualties. This accommodation proved of inestimable value during subsequent air raids and enabled the Hospital to carry on its work despite the damage it had sustained.

The Children's Hospital received extensive damage as the result of a stick of bombs falling across the building, the out-patient department, and the Nurses' Home. One very large bomb fell in the garden, tore off the tiles, broke down the doors, and smashed nearly all the glass in the windows. The electric light was extinguished, as were the emergency lights, the night lights and hurricane lamps, plunging the whole building in total darkness. In the midst of all this damage, with the plaster falling from the ceilings and the crashing of falling masonry all the patients were evacuated without a single casualty and taken to another Hospital.

The corridors were a mass of broken glass, plaster and debris, over which the children had to be carried, while bombs constantly exploded nearby. Many times both nurse and patient had to lie on the ground to avoid blast, the nurse covering the child with her body. All this was done in complete darkness. The behaviour of the Matron, Miss G. R. Ellis, and nurses was beyond all praise, and they have been presented with a framed appreciation by the Lord Mayor on behalf of the Committee.

The next day about thirty soldiers were sent to the Hospital to help clear away the tremendous quantity of debris, which included not only broken masonry but an immense quantity of soil and clay thrown up by the explosion. These men worked for days in an endeavour to get the wards, corridors and out-patient departments free of rubbish. In addition, the City Engineer sent a large number of workmen to undertake first-aid repairs to the building. The damage was so extensive that it took nearly three months to get the Hospital into a condition to receive patients. The out-patient department was so damaged by a direct hit, that it has been abandoned for this purpose.

The Nurses' Home also received a direct hit, the bomb falling into a 500-gallon water tank under the roof. This tank, which is one of several, was destroyed, but it caused the explosion to be upward, tearing off the tiles, smashing windows and tearing off the plaster. It was simply miraculous that with all this destruction there were no casualties. It seemed hopeless to get the Hospital into order again, but by the united efforts of the City, soldiers and the staff themselves this was achieved, and the Bristol Children's Hospital resumed its work of treating boys and girls who were ill.

The Southmead Hospital escaped heavy damage by near misses of bombs. A parachute with a basket of incendiaries was sighted in its descent. By skilful operation of the staff, the threatened conflagration was averted and the parachute salvaged intact as a souvenir. The fabric was of white artificial silk, beautifully made, with its markings of German manufacture, cords, loops and rings all intact. In one raid, high explosives fell. One was in close proximity to Dr. Phillips' residence, where groups of nurses were taking shelter. The sub-soil of the site is soft clay to a considerable depth. The bomb penetrated deeply and its explosion raised a huge blister or mould of unbroken turf. The clay, acting as a cushion, had absorbed the shock, disrupting the underground cables and pipes with no damage to the adjacent building or injury to the staff.

The reception of air-raid casualties was spread over 25 days and nights following the visits of the enemy over a period of six months. The casualties admitted were 410, the highest number on one day being 81. A wonderful spirit and great fortitude prevailed amongst all the patients, as in all the Hospitals. Sallies of humour often brightened the wards as the patients gained strength in body and spirit. The Lord Mayor and Lady Mayoress, accompanied by Mr. C. H. W. Davey (the Sheriff of Bristol) and Mrs. Davey, on one occasion were greeting a young man of 20 years very ill with chest and ankle injuries. He brought smiles to all when he said chirpily to the Lady Mayoress, "I like your hat." Then the child from Australia, who declared he came from "down under," focussed the amused attention of the group around his cot upon the Lord Mayor whose name had been thus inverted. This brave little fellow had lost his father in

Australia and his mother and grandmother were slain by the Nazis at Avonmouth. Now resident in Edinburgh, he writes regularly to his Ward Sister.

A young married couple had each lost a leg, amputated, but they were jolly and bright about it all. During their convalescence they playfully teased each other and laughed over the length of stump each had left.

A father and mother in separate wards had their five children, aged 4, 6, 8, 10 and 12, in an adjacent ward. They were a remarkable family and shed happiness around them. The four elder children sang solos and a chorus to the Lord Mayor. Their name was Plenty, an appropriate epithet where there was such an abundance of mutual love and happiness shown between parents and children.

At another visit two married ladies, sisters, in adjacent cots, greeted the Lord Mayor with charming smiles to reveal to him they were his former pupils. Fortunately the press photographer was awaiting his opportunity, so the cots were placed together and the Lord Mayor sat upon the bedside renewing old acquaintances whilst the camera recorded the re-union. These incidents may each appear to be trivial, but amidst the scenes in the Hospital such bright patches were turned to the best psychological use, as the hand-maiden to skilful surgery, under the inspiring leadership of Dr. Phillips and Miss E. Webber the Matron.

Attached to this Hospital is a Blood Transfusion Unit. Many lives were saved by this system. Altogether 530 pints of blood were transferred to 204 casualties in Bristol Hospitals. Of these, 227 pints of blood were used in Southmead Hospital for 82 patients. It is estimated that from 120 to 150 Bristol lives were saved through the operation of blood transfusion.

At the Cossham Memorial Hospital over 100 casualties were admitted from seven air raids and a large number of out-patients were also treated. The devotion to duty and the courage of the nursing personnel was an inspiration on all occasions. The patients displayed great fortitude, those less seriously injured cheering and helping their comrades. The injured from a particular factory were magnificent in spite of terrible injuries and burns and they literally would not take their plight seriously. One remarkable little patient was a child aged two-and-a-half years. Both her parents had been killed and she quickly became the mascot of the Hospital. On many occasions she was seen in the City escorted and being treated to tea by a member of the nursing staff off duty. The seriousness of her injuries was belied by the cheery piping voice and the sunny smile.

The Bristol Royal Infirmary received heavy air-raid damage ; on different occasions windows and frames were blown away, steam mains, lifts and the lighting and power systems were put out of action, the mortuary, the mortuary chapel with its beautiful stained-glass windows, the Calorifier House machinery, the out-patients department, the pathological, fracture and massage departments were all seriously damaged with their valuable contents. High explosives caused the evacuation of the Nurses' Home. The estimated cost of the damage exceeded £20,000, yet the Hospital functioned effectively and the staff bravely carried on amidst the dangers and under the grave handicaps to their life-saving work. About 500 casualties were admitted and well over that number of out-patients were dealt with. In addition, patients were received from the Eye Hospital which was evacuated when threatened by surrounding fires ; 50 more patients were admitted from the General Hospital when that was hit.

To provide for possible contingencies many patients were evacuated to the Royal West of England Sanatorium at Weston-super-Mare, the Birmingham Hospital Saturday Fund Home at Kewstoke, and Eastern House School. Distributions of fracture and other patients were made to St. Martin's Hospital at Bath, and to the Winford Orthopædic Hospital at Winford. St. Monica's Home gave up half their number of beds to replace those lost by the Bristol Royal Infirmary. In those evacuations ambulance drivers, voluntary drivers and owners of private cars gave splendid service.

In the absence of power one night the lifts were manned by volunteers until 3 a.m., when the Army Traffic Control arrived and took over the work of the already tired stretcher bearers. The staff worked by candle and torch light during one raid when all lights were fused. They were themselves fortified in their courage by the example of Auxiliary Fire Service men who came in as many as six times for dressings and went back to duty although their hands were badly burned.

In the later months of this tragic period America sent large quantities of clothing which was used for re-fitting families. Supplies of surgical instruments and other gifts from America replaced much of the destroyed equipment.

The Bristol Hospitals which received casualties were visited by the Lord Mayor and Lady Mayoress a few days after each blitz. They stood by the bedside of over 1,000 citizens in their 36 visits to these Hospitals and whenever possible gave to each one a message of comfort and cheer, expressing the admiration of their fellow citizens for the courage the patients had displayed as casualties in the front line of the battle for liberty. Never was there one word of complaint from those brave men, women and children ; from their lips and their hearts came words of loving gratitude for the wonderful deeds of bravery, kindness and skill shown them in the time of their suffering. The Lord Mayor publicly testified on many occasions how deeply he had been affected by those bedside talks and how highly he appreciated the magnificent skill and integrity shown by all members of the Hospital Staffs in this City.

## Figures and Tables

**Figure f1:**
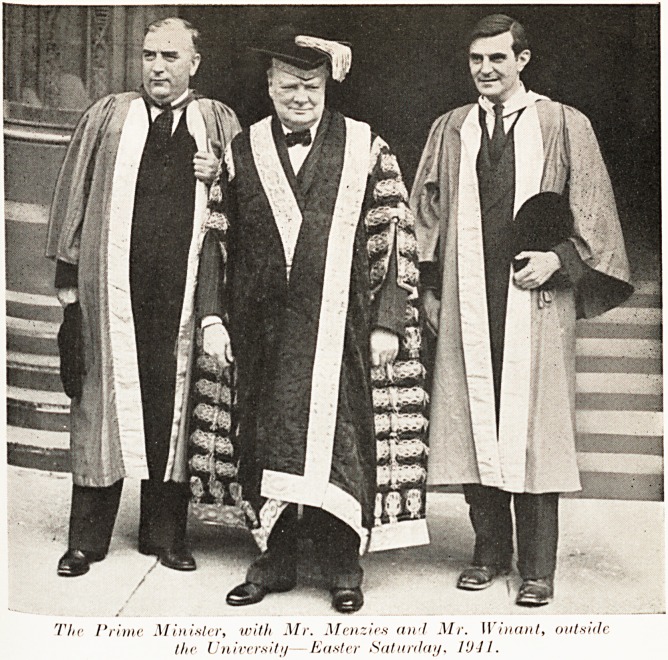


**Figure f2:**
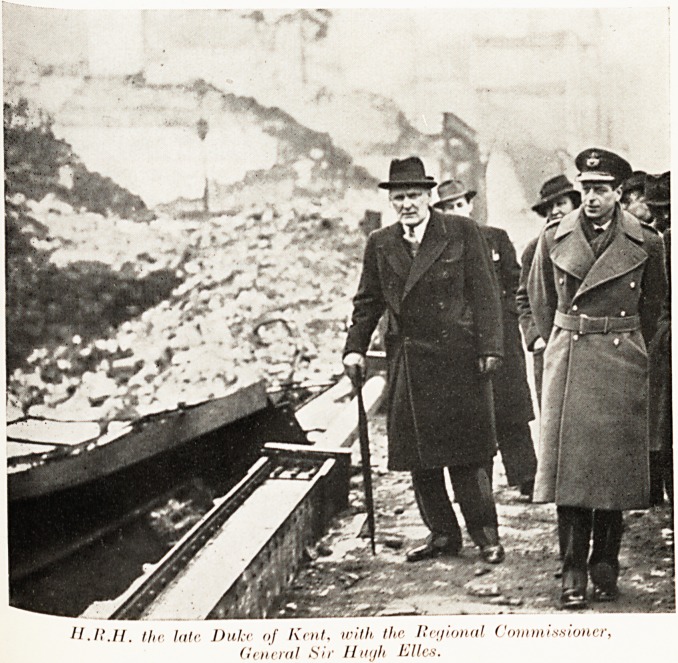


**Figure f3:**